# Type A and Type D Personality in Cardiovascular Health: A Narrative Review

**DOI:** 10.3390/healthcare14142199

**Published:** 2026-07-21

**Authors:** Diana Mariana Banceu, Horatiu Suciu, Cosmin Marian Banceu

**Affiliations:** 1 Organizing Institution for Doctoral University Studies, West University of Timisoara, 300223 Timisoara, Romania; 2 Department of Psychology, George Emil Palade University of Medicine, Pharmacy, Science and Technology, 540142 Targu Mures, Romania; 3 Department M3, George Emil Palade University of Medicine, Pharmacy, Science, and Technology of Targu Mures, 540139 Targu Mures, Romania; horatiu.suciu@umfst.ro; 4 Emergency Institute for Cardiovascular Diseases and Transplantation Targu Mures, 540136 Targu Mures, Romania; cosmin.banceu@umfst.ro; 5 Department ME 2, George Emil Palade University of Medicine, Pharmacy, Science, and Technology of Targu Mures, 540139 Targu Mures, Romania

**Keywords:** Type A personality, Type D personality, cardiovascular diseases, risk factors, cardiac surgical procedures

## Abstract

This narrative review critically summarizes the literature on the associations of Type A and Type D personality, together with related personality traits, with cardiovascular disease (CVD) onset, prognosis, cardiac surgery, and rehabilitation outcomes. For the past half a century, there has been a consistent interest in the question of whether or not there is a connection between personality qualities and CVD. At the same time as individuals with a Type A personality who were angry, competitive, and excessively motivated were overrepresented among patients seeking treatment for CVD, it was also noted that these individuals were more likely to acquire coronary artery disease or syndrome. Anger and animosity were among the unfavorable impacts that were found to be connected with worse cardiovascular outcomes, according to the findings of research. After that, a new personality entity was brought into existence, which was referred to as the type D “distressed” personality. This personality type coupled negative affectivity and social inhibition. Type D personality subsequently became a major focus of research, and several studies reported associations with poorer patient-reported health and adverse cardiac outcomes. However, these findings have not been consistently replicated, and the stability, incremental predictive value, and independence of Type D personality from depression, disease severity, and other psychosocial factors remain debated. As a result, there are a number of criticisms that pertain to the current knowledge of the connection between personality construct and the risk of developing cardiovascular diseases as well as the outcome of these diseases. This review provides a critical narrative synthesis of supportive and conflicting evidence and highlights the methodological limitations that currently prevent definitive causal or prognostic conclusions.

## 1. Introduction

Cardiovascular disease is the primary cause of mortality worldwide in comparison to other diseases. Psychosocial factors have been identified as important contributors to the manifestation and prognosis of cardiovascular diseases among the many risk factors assessed [1,2]. Moreover, amongst many psychological and social elements, an individual’s personality features or characteristics have been examined as a significant influence on the morbidity of cardiovascular disease (CVD). The relationship between personality and cardiac disease, namely coronary heart disease (CHD), has been examined in numerous research over the past 40 to 50 years [3]. Coronary heart disease is a medical condition characterized by the obstruction of coronary arteries due to atherosclerotic plaque accumulation. Numerous risk factors contribute to this disease, one of them being psychosocial issues. Individuals persistently exposed to psychosocial variables face an elevated risk of CHD. The correlation between personality type and the risk of heart disease exists, although the significance of personality in relation to heart disease remains contentious [4].

Personality encompasses an individual’s cognitive processes, emotional states, and behaviors that influence health outcomes. Type D and type A personalities are considered potential risk factors for CHD [5,6]. Prior research concentrated on personality typologies and identified Type A personality—characterized by antagonism, impatience, and competitive dominance—as negatively correlated with the occurrence of cardiovascular diseases [3,7]. Type D personality is characterized by negative affectivity and social inhibition and has been investigated as a potential psychosocial correlate of prognosis in patients with CHD [8]. Type D personality exhibited a strong correlation with plaque susceptibility, while Type A personality did not [9]. Research on Japanese populations indicated that Type A personality did not forecast CHD events, while Type B behavior patterns in men were associated with a heightened risk of CHD [10]. This finding suggests that the specific personality characteristics associated with an increased risk of CHD remain inadequately understood.

The role of personality type as a predictive factor for CHD was assessed via biological and behavioral mechanisms [8]. Coping is a behavioral mechanism anticipated to elucidate the impact of personality types on CHD. Coping encompasses the strategies and responses employed by individuals to navigate a circumstance. Coping mechanisms are associated with medical, psychological, cultural, social, and individual experiences and traits. Research on personality types and coping methods indicated that individuals with type D personality employed maladaptive coping strategies when managing their illnesses.

Personality influences an individual’s response to psychological or emotional distress and may affect behavior relevant to health. Because CVD is frequently associated with modifiable behaviors, personality-related characteristics may be linked to disease onset and prognosis. Accordingly, this narrative review aims to critically examine the associations of Type A and Type D personality, together with related traits such as hostility, anger, neuroticism, conscientiousness, and optimism, with CVD onset and prognosis. It also evaluates their implications for cardiac surgery, implantable cardiac device therapy, and cardiac rehabilitation (CR) and discusses the biological, behavioral, and psychological mechanisms that may underlie these associations.

## 2. Materials and Methods

A methodical literature search of PubMed, Scopus, Web of Science, and Google Scholar was performed to conduct this narrative review. Combinations of personality, Type A and Type D personalities, hostility, neuroticism, Big Five qualities, cardiovascular disease, coronary heart disease, cardiac surgery, cardiac rehabilitation, anxiety, depression, and psychosocial risk were among the search terms. Peer-reviewed publications, systematic reviews, meta-analyses, cohort studies, and therapeutically relevant narrative reviews published in English were taken into account. When historically relevant, foundational documents were kept, but the most recent evidence was given priority when it required interpretation. The evidence was narratively summarized based on cardiovascular outcomes, processes, and characteristics of personality.

### 2.1. Review Design

This article was designed as a narrative review rather than as a systematic literature review or meta-analysis. A structured literature search was used to support the narrative synthesis without claiming exhaustive identification of all eligible publications. Accordingly, this review was not preregistered, and the PRISMA framework was not applied. To enhance transparency, the databases, search concepts, eligibility criteria, publication types, and selection approach are reported below.

### 2.2. Information Sources and Search Strategy

PubMed, Scopus, Web of Science Core Collection, and Google Scholar were searched from database inception to conduct this narrative review. Search concepts were organized into personality-related and cardiovascular-related blocks. The principal combination was (“personality” OR “Type A personality” OR “Type D personality” OR hostility OR anger OR neuroticism OR “Big Five” OR conscientiousness OR optimism) AND (“cardiovascular disease” OR “coronary heart disease” OR “coronary artery disease” OR “cardiac surgery” OR “coronary artery bypass graft*” OR “cardiac rehabilitation”). Additional searches combined these concepts with anxiety, depression, coping, psychosocial risk, health behavior, prognosis, mortality, and quality of life. Reference lists of relevant reviews and landmark studies were also screened for additional publications.

### 2.3. Eligibility Criteria

**Figure 1:**
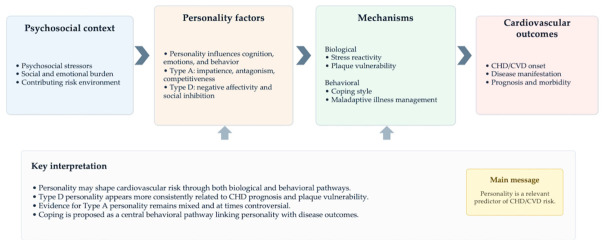
Schematic illustration of the relationships among psychosocial context, personality factors, biological and behavioral mechanisms, and cardiovascular outcomes.

English-language, peer-reviewed publications addressing personality constructs or traits in relation to cardiovascular risk, CVD, cardiac outcomes, cardiac surgery, or CR were considered eligible. Empirical observational and interventional studies were used to support statements concerning associations, biological and behavioral mechanisms, prognosis, quality of life, and intervention outcomes. Systematic reviews and meta-analyses were used to summarize broader patterns of evidence and inconsistency. Conceptual, theoretical, instrument-development, and historically important publications were included only to define personality constructs, describe their development and measurement, and inform the conceptual framework shown in Figure 1; they were not treated as empirical evidence of cardiovascular associations or outcomes.

Publications were excluded when they did not assess a personality construct or related trait, did not address a cardiovascular or clinically relevant cardiac outcome, were not available in English, consisted only of conference abstracts, or duplicated data reported in another eligible publication. Studies addressing depression or anxiety without an explicit connection to personality and cardiovascular outcomes were also excluded. “Foundational publications” were defined as original or landmark articles that introduced the Type A or Type D constructs, described their principal measurement instruments, or provided early longitudinal or intervention evidence that substantially influenced subsequent research.

### 2.4. Publication Selection and Narrative Synthesis

Titles and abstracts were screened, followed by full-text assessment. Publications were grouped according to personality construct, cardiovascular outcome, proposed biological, behavioral, or psychological mechanism, and cardiac surgery. Because of substantial heterogeneity in study populations, personality instruments, study designs, and cardiovascular endpoints, no quantitative synthesis was undertaken.

## 3. Narrative Synthesis and Discussion

### 3.1. Personality Types and Cardiovascular Health

#### 3.1.1. Type A Personality and Cardiovascular Outcomes

The potential influence of personality on CVD was initially noticed by Friedman and Rosenman (1959) [11] in a cohort of individuals with coronary artery disease (CAD). Individuals exhibiting Type A personality traits have been shown to be susceptible to heightened stress levels and additionally at an increased risk for CAD. Furthermore, a robust correlation was identified between Type A behaviors and blood cholesterol levels, blood coagulation time, occurrence of arcus senilis, and clinical CAD [11]. Subsequently, it was suggested that individuals exhibiting Type A personality traits faced a heightened risk of CAD and other causes of early mortality, even when accounting for other risk factors [12]. Moreover, the same authors proposed that modifying Type A behavior patterns during myocardial infarction may reduce the eventual incidence of reinfarction [13]. Although numerous research indicates a robust association between Type A personality traits and CVDs, the potential influence of Type A personality on cardiac conditions has been re-examined. Subsequent research assessing this potential connection revealed no substantial linkage or relationship among the two [14,15]. A 2002 systematic analysis, which synthesized the results of 18 etiological and 15 prognostic studies, indicated that studies demonstrating a significant link were in the minority in both categories [16]. Following studies has demonstrated no correlation with mortality: for instance, the Prospective Epidemiological Study of Myocardial Infarction (PRIME) study, which investigated psychosocial risk factors for CVD in France and Northern Ireland [17]; the GAZEL study, which revealed no link between Type A behavior and mortality in French men, and identified it as protective against all-cause mortality in women [18]; and the Japan Public Health Center-based Prospective Study (JPHC), which indicated that Type A behavior was not predictive of CHD in a Japanese cohort [10]. A meta-analysis of 25 prospective studies on CAD and Type A personality did not reveal a significant association between the two. In contrast, a significant relationship was identified among hostility and the risk of CAD (n = 15,038), indicating a potential and substantial role of anger and hostility in CAD and CVD [19]. Consequently, subsequent investigations redirected their investigations attention from the specific Type A behavior pattern to antagonism as a contributing factor in the development of CVD. Numerous research indicate that the hostility and anger elements of the Type A behavior pattern are more reliable indicators of CVD [19,20,21]. A meta-analysis of 25 studies, along with a separate analysis of 19 studies examining CHD outcomes in healthy individuals and those with preexisting CHD, demonstrated that both anger and hostility were significantly correlated with a higher risk of CHD occurrences in both populations [20].

Type A personality people exhibit physiological features such as elevated noradrenaline levels, accelerated blood-clotting times, increased cholesterol levels, and heightened plasma lipid levels, including triglycerides and low-density lipoprotein cholesterol [22,23,24]. Sensitivity to cortisol correlates with hostility [25], while plasma c-reactive protein (CRP) levels exhibit a negative correlation with Type A personality [26]. Type A individuals are frequently observed to pursue challenging and competitive environments, exhibiting higher rates of smoking and alcohol consumption compared to Type B individuals [27]. This increases their risk of CVD. Individuals often underreport the severity of their physical symptoms, potentially leading to untreated disease progression. Type A behavior is considered a significant risk factor for physical disorders. Individuals in this group exhibit a higher likelihood of experiencing accidents, mortality due to accidents or violence, and the incidence of CVD [28].

#### 3.1.2. Type D Personality and Cardiovascular Outcomes

Type D personality is also termed as ‘distressed’ personality [29,30]. Following the seminal research on decadal mortality post-myocardial infarction [31], Type D personality was associated with outcomes in various cardiac patient cohorts. Type D individuals exhibit a heightened risk for diminished quality of life and appear to derive less benefit from medicinal and invasive interventions [32]. Research indicates that individuals with Type D personality and CVD exhibit a greater prevalence of cardiac symptoms yet are less inclined to communicate these symptoms (e.g., oedema, dyspnea) to healthcare providers or to seek medical attention promptly, leading to exacerbated conditions due to their propensity for social inhibition [33]. Moreover, individuals with Type D personality are reported to have heightened illness severity [34], elevated cardiac mortality [35], compromised health status, and higher depressed symptoms [36]. Type D personality has been associated with both fatal [32] and nonfatal (noncardiac chest pain) incidents [37,38] and exhibits a higher incidence of work-related issues [39]. Negative affectivity and social inhibition, which characterize Type D personality, are associated with neuroticism [40]. Neuroticism is similarly linked to adverse health outcomes, cardiovascular risk factors, and CHD. Significant intersect in the polygenic architecture among genes related to neuroticism and those associated with CHD and CVD risk factors indicates that genetic factors may partially contribute to the relationship among neuroticism and cardiovascular dysfunction [41].

Several significant molecular pathways have been identified as underlying the association among Type D personality and CVD. Type D personality is correlated with heart rate and heart rate recovery in males. Diabetes mellitus, a known risk factor for CHD, exhibited a correlation with anger, while fasting blood sugar levels were linked to extraversion. However, no associations were found regarding duration D [42]. These were derived from certain biological discoveries: In patients with heart failure, Type D personality significantly predicts elevated circulation levels of the proinflammatory cytokine tumor necrosis factor, which is related with the development of heart failure [43,44]. Individuals exhibiting Type D personality demonstrated elevated cortisol-awakening responses, irrespective of demographic and clinical variables as well as depression, indicating a potential link between Type D personality and dysregulation of the hypothalamic–pituitary–adrenal axis, which may contribute to the etiopathogenesis of coronary artery disease [45,46]. Individuals with Type D personality exhibit heightened blood pressure and cardiac responses to stressful events, thus elevating the chance of developing CVD or CAD [47,48,49]. Moreover, research indicates that persons with CVD exhibiting Type D personality demonstrate diminished cognitive functioning, independent of depression and anxiety [50]. Type D personality, Type A personality, extraversion, and anger are correlated with metabolic conditions, including body mass index, waist circumference, triglycerides, and blood pressure [26,51,52,53]. Additionally, optimism exhibited a direct correlation with CAD [54].

Individuals with Type D personality are more likely to employ maladaptive coping strategies, namely utilizing less confrontational coping and more accepting resignation coping in reaction to CVD. Furthermore, confrontation coping was identified as a mediator in the relationship between Type D personality and perceived disease severity, while acceptance–resignation coping mediated the connection between Type D personality and morale. This indicates that coping modification strategies should be a crucial component of psychological interventions for individuals with CVD and Type D personality [55].

Research indicates that individuals with CVD prior to implanted cardioverter defibrillator (ICD) installation exhibited a Type D personality pattern, which correlated with poorer physical and mental health status [56]. Additionally, research indicates that patients with ICD exhibiting a Type D personality experience heightened depression symptoms when partnered with others of the same personality type [57]. Research indicates that individuals with Type D personality who undergo percutaneous coronary interventions (PCI) exhibit an approximately 3.69-fold heightened risk of depression and a 2.72-fold elevated risk of anxiety after 10 years of follow-up [58]. Consequently, research has also aimed to examine the influence of the psychological profile of carers or partners of patients with cardiovascular diseases.

Adherence to treatment recommendations encompassed medication adherence, dietary adherence, compliance with sports recommendations, self-care practices, and consultations with health practitioners—these factors were associated with Type D personality traits, including openness, conscientiousness, optimism, neuroticism, and agreeableness, but did not show a positive correlation with positive affect [52,59]. Dietary behaviors, including the intake of unhealthy foods and fruit, were associated with Type D personality, conscientiousness, and anger, whereas only conscientiousness was linked to exercise [51,60]. Smoking exhibited associations with Type D personality, conscientiousness, and neuroticism, while showing no correlation with Type A personality [52,61,62]. Type D personality, negative affect, and hostility are associated with alcohol use [52,63,64]. Personality traits linked to positive health behaviors include conscientiousness, openness, and agreeableness, whereas extraversion is associated with negative health behaviors [60]. These dietary behaviors may serve as contributing factors to CVDs.

### 3.2. Personality-Cardiac Disease Link

#### 3.2.1. Biological, Behavioral, and Psychological Mechanisms

Personality types contribute to CHD through three primary mechanisms: behavioral, psychological, and biological. The behavioral mechanism indicated that personality affects health by promoting less healthy behaviors and inhibiting actions that could enhance health [65]. Certain individuals may exhibit a functioning style marked by heightened neuroendocrine and sympathetic responses to perceived stressors. Individuals in this category perceive challenging situations as more threatening than their counterparts and exhibit heightened physiological responses. Personality influences health indirectly by contributing to excessive health-degrading behaviors and a deficiency in health-promoting behaviors. Certain personality traits, including conscientiousness and openness to experience, have been linked to health-promoting behaviors that mitigate the risk of medical health issues [66]. Additionally, a noncausal association between personality and health has been proposed, indicating that an underlying genetic or constitutional factor may lead to both physiological vulnerability to disease and the behavioral, emotional, and cognitive traits associated with personality [66].

It has been reported that the association between Type A personality patterns and cardiovascular diseases is not definitively established due to inconsistent findings [19,67]. Since 2000, the new D personality type concerning cardiac sickness has become the subject of research. Two distinct meta-analyses of 10 prospective studies and 15 studies, respectively, investigating the association between Type D personality and health status in patients with CVD, indicated that Type D personality should be considered an independent correlate of diminished patient-reported physical and mental health status [68,69]. Moreover, the cross-cultural validity of the Type D construct and its association with CVD has been examined in 22 countries involving approximately 6222 patients, demonstrating a universal correlation between Type D personality and certain cardiovascular risk factors, thereby affirming the significance of Type D personality across diverse cultures and nations [70]. Nonetheless, regardless of extensive research in this domain, the utility of Type D personality in elucidating the connection between behavior and cardiovascular disorders has been challenged by certain scholars, and numerous enquiries have emerged regarding the meta-analyses of Type D personality studies [71].

Individuals with Type D personality are associated with unhealthy lifestyle choices, including reduced physical activity, a less varied diet, inadequate fat intake regulation, and low adherence to health guidelines, resulting in poorer outcomes for Type D patients [72]. Inappropriate health behaviors contributing to obesity, metabolic dysfunction, and endothelial dysfunction can result in hypertension, diabetes mellitus, dyslipidemia, inflammation, and thrombosis, all of which are risk factors for CHD.

Experts have posited that Type D personality constitutes a variant of neuroticism and interrogated what supplementary psychological risk factor is acquired by examining Type D personality [73]. Moreover, apprehensions have been expressed about the emphasis on singular personality traits, such as Type D, which diminishes the significance of mood states like depression, anxiety, and vital exhaustion in the etiology of CVD, all of which were evaluated as independent risk factors [74,75].

Moreover, Type D personality has been identified as a susceptibility sign that impacts individuals with CVD as well as various other medical conditions [76]. It is currently considered a risk factor for psychological discomfort and is linked to disease-promoting pathways in healthy individuals [76]. Consequently, attributing Type D personality characteristics exclusively to cardiovascular diseases is unwarranted.

The study and measurement of the effects of psychosocial factors on CHD present significant challenges due to the considerable variability and case-specific differences of these factors [2]. Psychological stress conditions activate the sympathetic nervous system, which regulates heart rate, catecholamine release, and the dysregulation of the hypothalamic–pituitary–adrenal axis (HPA) [77]. Type D personality represents an early stage of CAD [78] and is linked to anxiety and depression. The relationship between personality type and anxiety in populations with coronary heart disease was significant [79]. Furthermore, depression is associated with the stability of atherosclerotic plaque. Exposure to psychosocial factors consistently influences unhealthy behaviors, serving as a mechanism through which these factors indirectly affect CHD [80].

A meticulous examination of the literature about Type D personality and its correlation with CVD reveals that most of the research originated from a singular institution and a specific group of researchers [81,82]. Research has not identified a predictive value for Type D personality [83], leading researchers to propose that earlier studies may have exaggerated its prognostic significance [84]. Moreover, it has been determined that additional methodologically rigorous investigations are necessary to establish any definitive conclusions regarding the relationship between the personality construct and its predictive relevance for cardiovascular diseases [84]. Kupper and Denollet (2016) revealed that Type D personality is linked to a heightened risk of cardiac events; however, they also found that Type D personality was not connected with noncardiac mortality or events in those over 70 years of age [85]. Few studies have challenged the validity of Type D personality, revealing that conscientiousness and the big five personality traits more accurately predict health-related variables and behaviors [86]. Conversely, other research has affirmed the predictive significance of Type D personality concerning impaired endothelial function in individuals with CAD, indicating that those with Type D personality exhibit a heightened risk of endothelial dysfunction [87]. A hybrid personality construct combining Type A and Type D typologies has been found in a cohort of individuals with essential hypertension and acute coronary syndrome. The six aggregated personality profiles discovered through cluster analysis were Type D, Type A adversely impacted, not Type A negatively impacted, socially inhibited–positively impacted, not socially inhibited, and neither Type A nor Type D. The Type A negatively impacted cluster had the most adverse cardiovascular profile, indicating the necessity for a novel method to detect integrated personality profiles [6].

Negative affectivity, a facet of Type D personality, significantly coincides with the concept of depression. The inquiry into whether Type D is a genuinely stable personality rather than a reaction to illness necessitates additional elucidation, as many studies have evaluated this personality construct in patients already diagnosed with CVD. The awareness of the condition may result in adverse mood states and constraints in social relationships.

#### 3.2.2. Clinical Implications of Personality Categories in Cardiovascular Disease Patients

The hypothesis posits that personality influences the development and outcomes of CVD and CAD, leading to investigations into that altering personality traits and behavioral patterns can mitigate risk and diminish adverse outcomes associated with CVD [8]. Prior research in the Recurrent Coronary Prevention Project concentrated on modifying Type A behavior patterns and negative affect through group therapy. Findings indicated that participants who engaged in behavioral alteration counselling experienced a notable decrease in cardiovascular mortality and nonfatal myocardial infarction rates [13]. Limited research exists that has pursued behavioral change in individuals with CVD and concomitant Type D personality. A randomized controlled trial aimed at modifying Type D behavior split patients into enhanced cardiac rehabilitation and standard rehabilitation [88]. The supplementary therapies correlated with enhanced quality of life and a nonsignificant improvement in Type D scores, depression, and anxiety. Research has demonstrated the positive impact of cardiac rehabilitation programs, when integrated with relaxation and meditation techniques, on depressed and anxiety symptoms in patients with CVD [89]. Consequently, these findings indicate potential for interventions to alter coronary-prone behaviors; nevertheless, additional research is necessary before definitive inferences and conclusions can be established.

#### 3.2.3. Cardiac Surgery Outcomes and Psychological Risk

Surgery serves as a significant source of diverse physical and psychological stimulus for patients. Surgery can induce anxiety, fear, pessimism, and other negative emotions for those who have not fully processed the emotional impact of their illness. This subsequent anxiety is generally recognized as a typical reaction among preoperative patients [90,91]. Stress associated with surgery can result in negative emotions for certain patients, potentially impacting postoperative outcomes. Prior research indicates a significant correlation between anxiety and health outcomes across different patient groups [92,93,94,95]. Preoperative anxiety associated with anesthesia is a significant challenge for numerous patients [96]. Preoperative anxiety is a significant concern for anesthesiologists, as it is recognized as a risk factor for perioperative complications.

Preoperative anxiety was associated with state anxiety, while personality characteristics like neuroticism and Type A personality were linked to trait anxiety. Additionally, there was a strong correlation between state and trait anxiety, suggesting that people with elevated levels of trait anxiety tend to experience heightened state anxiety in anxiety-inducing contexts, such as surgery. People exhibiting Type A personality characteristics are often highly competitive, self-critical, prone to stress, and likely to overreact. For patients with Type A personality, the waiting period before surgery can feel excessively prolonged and distressing [97].

Despite the expectation that coronary artery bypass graft surgery (CABG) would alleviate symptoms associated with ischemic heart disease, a few patients continue to experience these symptoms six months post-surgery. It is established that the majority exhibit Type D personality traits [98]. Mali et al. (2021) found that patients with CABG who have Type D personality exhibited considerably reduced quality-of-life scores compared to those without Type D personality across physical, psychological, and environmental domains [99]. Al-Ruzzeh et al. conducted a study indicating that Type D personality is a predictor of diminished physical quality of life following cardiac surgery [32]. However, other studies indicated that the quality of life was optimal 6–12 months post-surgery, encompassing the physical domain [100]. The frequency of depression decreases in older patients exhibiting Type D personality following cardiac surgery. This could stem from insufficient understanding of surgical risks and their prognoses. Conversely, older individuals may experience reduced concern and stress regarding functional loss and diminished performance post-surgery compared to younger patients. The detrimental impact of Type D personality on quality of life following CABG surgery has been established in the literature. It is important to recognize that this is distinct from depression, which may be linked to Type D personality [100]. Screening cardiovascular patients for Type D personality may reduce depression following CABG surgery [101]. Dannemann et al. (2010) [102] demonstrated that Type D diagnosis altered in approximately 60% of cases following surgery. Only patients with stable Type D demonstrated a correlation with emotional distress, including anxiety and depression, as well as a diminished quality of life. It can be posited that Type D personality may undergo changes following significant life events such as cardiac surgery [102]. Matsuishi et al. (2019) [103] demonstrated that Type D personality serves as a predictive indicator for long-term acute brain dysfunction (delirium/coma) in cardiovascular patients undergoing surgery, independent of depressive symptoms. Furthermore, depressive symptoms linked to Type D personality exacerbate the severity of acute brain dysfunction [103].

### 3.3. Cardiac Recovery and Rehabilitation

Recognizing if heart failure patients with Type D personality traits are at an increased risk for ongoing worsening in their health-related quality of life (HRQoL) is crucial from a clinical standpoint. This insight could facilitate more precise risk stratification for heart failure patients entering rehabilitation programs and enable early interventions targeting behavioral and psychological risk factors to enhance patient outcomes.

The D type personality is linked to inflammation and may elevate mortality and morbidity associated with cardiovascular disease. It is recognized as a psychosocial risk factor for cardiovascular disease (CVD) [104]. Cardiac rehabilitation (CR) has the potential to enhance functional capacity, endurance, quality of life, and depression scores, irrespective of Type D personality traits. A carefully designed extensive cardiac rehabilitation program is associated with improvements in functional status, health-related quality of life, and reductions in mortality and depression scores in cardiovascular disease [105,106,107]. One study indicates that Type D personality is associated with diminished health-related quality of life (HRQoL) in patients undergoing cardiac rehabilitation for cardiovascular disease, both prior to and following CR [108]. Type D personality has been associated with diminished health-related quality of life (HRQoL) in patients with coronary artery disease (CAD) undergoing cardiac rehabilitation [109], and in those with chronic heart failure [110]. A prospective study indicated that CAD patients exhibiting Type D personality had a twice increased risk of reporting poor perceived health at the 5-year follow-up compared to non-Type D CAD patients [111]. Mental distress and perceived social support may partially explain the relationship between Type D personality and health-related quality of life (HRQoL). A potential reason for the decreased HRQoL in Type D personality patients is their increased risk of poor medication adherence, which may result in adverse health outcomes [112]. Patients with Type D personality exhibit anxiety and depression, leading to a tendency to avoid the expression of relevant and timely emotions. Suppressing emotional expression may diminish the quality of interpersonal relationships and health-related quality of life (HRQoL). The influence of Type D personality on HRQoL was significant, with only a slight reduction observed whenever signs of anxiety and social support were incorporated into the models, while depression symptoms did not have an effect. This frequently indicates the presence of effects attributable to more than one omitted mediator [113]. A study investigated health-related quality of life (HRQoL) in patients who underwent coronary artery bypass graft surgery six months post-operation. The findings indicated that heightened anxiety significantly mediated the effect of Type D personality on the lack of improvement in HRQoL. In contrast, increased depressive symptoms accounted for the decline in HRQoL independently of Type D personality influences [114]. Another investigation indicated that Type D patients are more likely to benefit from social interaction, such as group therapies [115].

Patients with Type D personality require focused attention during rehabilitation, as research indicates that this personality type independently predicts a reduction in health-related quality of life (HRQoL). Broadened cardiac rehabilitation decreases Type D scores, alleviates anxiety and depressive symptoms, and enhances quality of life [88].

## 4. Strengths and Limitations

This narrative review has several strengths. It integrates evidence on personality typologies and dimensional traits with biological, behavioral, and psychological mechanisms, clinical cardiovascular outcomes, cardiac surgery, and CR. It also considers both foundational and recent publications and presents supportive and conflicting findings, thereby offering a balanced overview of the clinical relevance and continuing controversies surrounding Type A and Type D constructs.

Several limitations should be acknowledged. Because this is a narrative rather than a systematic review, no preregistered protocol, formal risk-of-bias assessment, or quantitative synthesis was undertaken, and study selection and interpretation may be susceptible to selection bias. Only English-language publications were considered. Heterogeneity in populations, personality instruments, cardiovascular endpoints, and adjustment for depression, anxiety, disease severity, and lifestyle factors limit direct comparison and precludes causal conclusions. Many studies rely on self-report, categorical cut-offs, and different personality scales; anger may also be classified differently across instruments. Psychological risk factors frequently co-occur, and comorbid depression may confound associations between personality dimensions and CVD outcomes. These limitations should be considered when interpreting the evidence.

## 5. Future Perspectives

Future research should explore the potential variability of health consequences associated with personality characteristics across different contexts. Research must also consider age, gender, ethnicity, and developmental processes in the relationship between personality and CVD. Furthermore, future research must consider that personality traits are dynamic and may evolve over time and future randomized studies should investigate whether adding structured psychological interventions to cardiac rehabilitation modifies the relationship between negative affectivity, social inhibition, and rehabilitation outcomes. Repeated dimensional assessments before, during, and after rehabilitation should determine whether changes in anxiety, depression, physical activity, treatment adherence, and HRQoL are accompanied by changes in Type D dimensions and whether these changes mediate or moderate clinical benefit. Consequently, interventions can be tailored to address individuals at risk, thereby decreasing the likelihood of adverse cardiovascular outcomes. While primary prevention poses challenges, enhancing public awareness regarding the influence of personality traits on cardiac health may prove advantageous. Strengthening secondary prevention is essential, achievable through the identification of maladaptive personality traits in individuals with CVD and the implementation of psychotherapy sessions aimed at modifying these traits to mitigate the risk of adverse cardiac events and outcomes.

## 6. Conclusions

Current evidence suggests that selected personality-related constructs are associated with cardiovascular health, but the strength, specificity, and clinical relevance of these associations vary across constructs and outcomes. Within the Type A pattern, anger and hostility appear to show more consistent associations with cardiovascular risk than the global Type A classification. Type D characteristics have been associated in several studies with emotional distress, poorer patient-reported health, suboptimal adherence, and some adverse cardiovascular or rehabilitation outcomes. However, prognostic findings are heterogeneous and may be influenced by depression, anxiety, disease severity, health behaviors, measurement instruments, and instability of categorical Type D classification. Type D should therefore be regarded as a potential marker of psychosocial vulnerability rather than as an established independent causal risk factor or a fixed clinical diagnosis. Personality assessment may complement established biomedical and psychosocial risk evaluation by identifying patients who could benefit from additional support, but it should not replace validated cardiovascular risk models or clinical judgment. Future work requires prospective designs, repeated dimensional measurement, standardized outcomes, and randomized trials testing whether psychologically enhanced cardiac rehabilitation improves distress, physical activity, health-related quality of life, and clinical outcomes.

## Data Availability

No new data were created or analyzed in this study. Data sharing is not applicable to this article.
